# Association between Perceived Neighborhood Environment and Walking among Adults in 4 Cities in Japan

**DOI:** 10.2188/jea.JE20090120

**Published:** 2010-07-05

**Authors:** Shigeru Inoue, Yumiko Ohya, Yuko Odagiri, Tomoko Takamiya, Kaori Ishii, Makiko Kitabayashi, Kenichi Suijo, James F Sallis, Teruichi Shimomitsu

**Affiliations:** 1 Department of Preventive Medicine and Public Health, Tokyo Medical University, Tokyo, Japan; 2 Department of Psychology, San Diego State University, San Diego, CA, USA

**Keywords:** active transport, neighborhood environment, physical activity, policy, walking

## Abstract

**Background:**

Recent research highlights the importance of environment as a determinant of physical activity; however, evidence among Japanese is sparse. The aim of this study was to examine the association between perceived neighborhood environment and neighborhood walking for multiple purposes among Japanese.

**Methods:**

We conducted a population-based, cross-sectional study of 1461 Japanese adults (age: 48.2 ± 14.1 years, men: 44.8%). Neighborhood environment and walking were assessed by a validated questionnaire. The odds ratio of active walkers was calculated in relation to environmental characteristics after adjustment for age, sex, and other potential confounders.

**Results:**

Participants were more likely to walk when they perceived that there was high residential density (odds ratio, 1.47; 95% confidence interval, 1.11–1.96), fair land use mix–diversity (1.37, 1.04–1.81), good walking/cycling facilities (1.56, 1.19–2.04), and attractive aesthetics (1.49, 1.14–1.95). Environmental factors associated with walking differed with respect to the purpose for walking. The environmental characteristics associated with walking for daily errands and with walking for commuting were similar, and included residential density and land use mix. Walking for leisure was associated with walking/cycling facilities, aesthetics, and traffic safety. Stratified analyses showed some sex-specific associations. Among women, there was an unexpected inverse association of leisure walking with both residential density and land use mix–diversity.

**Conclusions:**

The association between neighborhood environment and walking differed by walking purpose. The results were generally consistent with those of studies conducted in Western countries, except for the association of high residential density and good land use mix–diversity with less leisure walking in women. These results suggest possible targets for environmental interventions to promote walking.

## INTRODUCTION

Regular physical activity reduces the risk of mortality, and the incidence of cardiovascular diseases, diabetes, and some cancers.^1^^–^^3^ However, a large part of the population is not physically active in Japan and in many other countries.^4^^,^^5^ Thus, physical activity promotion is a public health priority.^6^ Data on physical activity determinants and correlates are needed as a basis for developing effective interventions. Many studies have focused on individual demographics and psychobehavioral factors.^7^ However, recent progress in research suggests that certain environmental characteristics, such as residential density, access to destinations, walking facilities, aesthetics, safety, and access to exercise facilities are related to physical activity.^7^^–^^13^ Interventions that target individuals have only a minimal impact on the physical activity levels of whole populations^14^^,^^15^; however, changes to the environment are believed to have a long-term and substantial impact.^16^

Although there is accumulating evidence on the association between physical activity and environment, the relevant studies have been mostly limited to Western countries, in particular the United States and Australia^12^; only a few have been undertaken in Japan.^17^^–^^19^ Evidence from study settings—including Japan—where the environment, culture, and physical activity patterns differ from those of Western countries, is thus valuable. Indeed, evidence from Japan could support or refute the generalizability of previous studies conducted in Western countries, and/or add new findings regarding associations between environment and physical activity. Also, data from Japanese are needed for the development of physical activity interventions in Japan.

We previously reported associations of environment with physical activity, using a convenience sample of Japanese adults.^18^ In that previous study, environmental characteristics were associated with physical activity, but the findings were limited by the use of simple measures that could not differentiate the purposes for walking. In the present cross-sectional study, we used a random community sample from 4 Japanese cities and measured walking as the outcome. Because environmental correlates are specific to the type and purpose of physical activity,^11^^,^^20^ the aim of this study was to examine environmental correlates of neighborhood walking and its components, including walking for daily errands, walking for leisure, and commuting on foot.

## METHODS

### Participants and data collection

This cross-sectional study was conducted from February 2007 through January 2008. A total of 4000 residents aged 20 to 69 years and living in 4 Japanese cities (Koganei, Tsukuba, Shizuoka, Kagoshima) were randomly selected from the registry of residential addresses and stratified by sex, age (20–29, 30–39, 40–49, 50–59, and 60–69 years), and city of residence, so that the sample included 2000 subjects of each sex, 800 subjects of each age category, and 1000 subjects from each city. As a result, the addresses of 100 subjects of a specific sex, a specific age category, and a specific city were obtained. Four cities were chosen so as to include various environmental conditions. Koganei is in the Tokyo metropolitan area and Tsukuba is a university town located 50 km northeast of Tokyo. Shizuoka and Kagoshima are located in central and western Japan, respectively, and are the capital cities of prefectures that include both urban and relatively rural areas. For data collection, a questionnaire was sent to and collected from participants via postal mail. To increase the response rate, invitation letters that described the content of the study were sent to all 4000 subjects 2 weeks before the survey. During the survey period, a call center was established to answer the questions of the subjects. Nonrespondents were mailed 2 additional requests to join the survey. If a participant submitted an incomplete survey, we asked that the survey be completed again. Ultimately, of the 4000 subjects identified, 1508 (37.7%) responded to the survey. After data cleaning, valid data were obtained from 1461 participants (final response rate: 36.5%). All participants signed an informed consent document before answering the questionnaire, and the study received prior approval from the Tokyo Medical University Ethics Committee.

### Assessment of perceived neighborhood environment

On the self-administered questionnaire, the Neighborhood Environment Walkability Scale–Abbreviated Japanese Version (NEWS–AJ) was used as the environmental measure.^21^^–^^23^ The NEWS questionnaire was originally developed in the United States to evaluate several neighborhood environmental factors believed to be related to physical activity undertaken for multiple purposes. It has been used in various countries.^24^^–^^26^ The NEWS–AJ consists of 54 questions that assess 8 neighborhood environmental factors: (1) residential density, (2) land use mix–diversity, (3) land use mix–access, (4) street connectivity, (5) walking and cycling facilities, (6) aesthetics, (7) traffic safety, and (8) crime safety. Several of these factors are related to the concept of walkability, which is the ability to walk from one’s home to nearby destinations. “Neighborhood” in this questionnaire meant the area within a 15-minute walk from a participant’s residence. A sample of the questions used is shown in the Appendix. Scores on the 8 subscales were calculated by using a standardized scoring manual.^27^ Higher scores indicate a more favorable environment for walking. The score for residential density was calculated as the sum of the weighted score of 5 items.^27^ Land use mix–diversity was based on the reported walking distance to a list of 23 possible destinations, including shops, services, and recreation facilities. As for the other variables, scores were estimated as the mean of scale items that used a 4-point rating scale (1 = strongly disagree, 4 = strongly agree), including reverse coding of selected items. The psychometric properties of the questionnaire and the process by which it was translated into Japanese were reported in a previous study.^23^ The test–retest reliabilities of the 8 subscales were from *r* = 0.76 to *r* = 0.96.

### Assessment of walking

For the assessment of physical activity, a self-administered questionnaire was used. The questionnaire asked participants about their walking frequency (days/week), and average walking duration each day (min/day), with respect to 6 purposes: walking for daily errands, walking for leisure, commuting on foot to work, commuting on foot to school, walking during work, and walking for other purposes. The questionnaire instructed participants to consider all walks that involved at least 5 minutes of continuous activity. Walking time (min/week) was calculated as the product of walking frequency and duration. In this study, 4 variables were examined: (1) neighborhood walking (sum of the duration of 4 types of walking, walking for daily errands, walking for leisure, commuting on foot to work, and commuting on foot to school, min/week), and 3 specific types of walking, namely, (2) walking for daily errands (min/week), (3) walking for leisure (min/week), and (4) commuting on foot to work (min/week). We examined these 3 specific types of walking because they were expected to occur in the participant’s neighborhood. Although commuting to school was also expected to occur in the neighborhood, we excluded this variable from the specific analyses because the present sample included only 31 participants (2.1%) who walked to school. The Spearman correlation coefficient between total walking time (the sum of 6 types of walking time) calculated from the questionnaire and step counts per day, as assessed by accelerometer in a part of the present study sample (*n* = 783), was 0.30 (*P* < 0.001).

### Sociodemographic and other variables

The sex and age of each participant were obtained from the registry of residential addresses of each city. Information on employment status, years of education, height, weight, and self-rated health was obtained by self-report. Body mass index (BMI) was calculated from self-reported weight and height. Self-rated health was measured with a single item that asked participants to rate their health: participants chose the most suitable answer from a 5-point scale—excellent, very good, good, fair, and poor—for the statement, “In general, would you say that your health is...?”.

### Statistical analyses

To examine the association between the neighborhood environment as the independent variable and walking as the dependent variable, odds ratios for active walkers were calculated using logistic regression models. For the analysis, the scores for the 8 environmental variables were converted into tertiles (high/middle/low for residential density and good/fair/poor for the other 7 variables). For each of the 4 walking variables, participants were classified into 2 groups. For neighborhood walking, participants were divided into 2 groups by using the median: ≤90 min/week or >90 min/week. Regarding walking for daily errands, walking for leisure, and commuting on foot to work, the proportions of participants who reported walking for these purposes were less than 50%. Thus, participants were divided into 2 groups for each of these purposes: those who walked for a given purpose and those who did not. In the analyses of commuting on foot to work, we used data only from employed participants (*n* = 1083). To calculate odds ratios, the environmental factors expected to be associated with lower levels of walking were used as references (“low” for residential density and “poor” for the other 7 variables), ie, an odds ratio higher than 1.00 indicates the association of an activity-supportive environmental characteristic with active walking. Odds ratios were adjusted by age, sex, location of residence, employment status, educational level, BMI, and self-rated health. Statistical significance was considered to be present when *P* < 0.05. All analyses were conducted by using SPSS version 15.0 for Windows (SPSS Inc., Tokyo, Japan).

## RESULTS

Table 1 shows the characteristics of the participants. In the overall sample, 44.8% were men. The mean age ± standard deviation (SD) was 48.2 ± 14.1 years. The sample included participants of Tsukuba (25.1%), Koganei (26.9%), Shizuoka (26.1%), and Kagoshima (21.9%). The proportion of overweight participants (BMI ≥25 kg/m^2^) was 26.5% of men and 12.4% of women. The proportions of participants who reported neighborhood walking, walking for daily errands, walking for leisure, and commuting on foot to work were 71.1%, 42.7%, 35.0%, and 29.0%, respectively.

**Table 1.** Characteristics of participants

	Overall		Men		Women	
	*n* = 1461		*n* = 654		*n* = 807	
			
	*n*	%	*n*	%	*n*	%
Age, years						
≤29	221	15.1	82	12.5	139	17.2
30–39	212	14.5	84	12.8	128	15.9
40–49	307	21.0	136	20.8	171	21.2
50–59	327	22.4	160	24.5	167	20.7
60+	394	27.0	192	29.4	202	25.0
mean ± SD	48.2 ± 14.1		49.6 ± 13.7		47.1 ± 14.3	
Location of residence						
Tsukuba	366	25.1	177	27.1	189	23.4
Koganei	393	26.9	172	26.3	221	27.4
Shizuoka	382	26.1	168	25.7	214	26.5
Kagoshima	320	21.9	137	20.9	183	22.7
Education, years						
≤12	600	41.1	268	41.0	332	41.1
13+	861	58.9	386	59.0	475	58.9
Employment status						
Employed	1083	74.1	559	85.5	524	64.9
Not employed	378	25.9	95	14.5	283	35.1
BMI, kg/m^2^						
≥25	273	18.7	173	26.5	100	12.4
<25	1188	81.3	481	73.5	707	87.6
Mean ± SD	22.4 ± 3.2		23.4 ± 3		21.5 ± 3.1	
Self-rated health						
Excellent	20	1.4	9	1.4	11	1.4
Very good	182	12.5	78	11.9	104	12.9
Good	577	39.5	245	37.5	332	41.1
Fair	603	41.3	281	43.0	322	39.9
Poor	79	5.4	41	6.3	38	4.7
Neighborhood walking^a^						
No	417	28.9	217	33.4	200	25.2
Yes	1026	71.1	432	66.6	594	74.8
Mean ± SD^b^, min/week	209 ± 185		203 ± 176		214 ± 191	
Walking for daily errands						
No	837	57.3	468	71.6	369	45.7
Yes	624	42.7	186	28.4	438	54.3
Mean ± SD^b^, min/week	121 ± 126		91 ± 101		134 ± 133	
Walking for leisure						
No	949	65.0	438	67.0	511	63.3
Yes	512	35.0	216	33.0	296	36.7
Mean ± SD^b^, min/week	180 ± 168		194 ± 180		170 ± 157	
Commuting on foot to work						
No	1038	71.0	426	65.1	612	75.8
Yes	423	29.0	228	34.9	195	24.2
Mean ± SD^b^, min/week	111 ± 90		123 ± 99		98 ± 76	
Commuting on foot to school						
No	1430	97.9	641	98.0	789	97.8
Yes	31	2.1	13	2.0	18	2.2
Mean ± SD^b^, min/week	106 ± 77		114 ± 83		101 ± 75	

^a^Neighborhood walking was defined as the sum of walking for daily errands, walking for leisure, commuting on foot to work, and commuting on foot to school.

^b^Mean ± SD indicates walking time for participants who did each type of walking.

Table 2 shows the mean scores and SDs for the 8 environmental variables. The tertiles of these variables are also indicated, and participants were categorized into 3 groups.

**Table 2.** Number and proportion of participants in each environmental category

	Range of category^a^	Overall		Men		Women	
		*n* = 1461		*n* = 654		*n* = 807	
		
		*n*	%	*n*	%	*n*	%
Residential density (5–805)^b^							
High	259<	432	29.8	178	27.5	254	31.8
Medium	184<, ≤259	514	35.5	234	36.1	280	35.0
Low	≤184	502	34.7	236	36.4	266	33.3
Mean ± SD		248 ± 96		242 ± 93		252 ± 98	
Land use mix–diversity (1–5)^b^							
Good	3.41<	471	32.8	214	33.3	257	32.4
Fair	2.57<, ≤3.41	483	33.7	211	32.9	272	34.3
Poor	≤2.57	481	33.5	217	33.8	264	33.3
Mean ± SD		2.95 ± 0.87		2.94 ± 0.84		2.96 ± 0.88	
Land use mix–access (1–4)^b^							
Good	3.14<	479	33.1	204	31.6	275	34.3
Fair	2.57<, ≤3.14	484	33.4	213	33.0	271	33.8
Poor	≤2.57	485	33.5	229	35.4	256	31.9
Mean ± SD		2.87 ± 0.63		2.85 ± 0.63		2.90 ± 0.64	
Street connectivity (1–4)^b^							
Good	3.00<	436	30.3	192	29.8	244	30.7
Fair	2.70<, ≤3.00	540	37.6	233	36.2	307	38.7
Poor	≤2.70	462	32.1	219	34.0	243	30.6
Mean ± SD		2.80 ± 0.73		2.76 ± 0.77		2.83 ± 0.7	
Walking/cycling facilities (1–4)^b^							
Good	2.40<	473	32.8	195	30.3	278	34.9
Fair	1.80<, ≤2.40	457	31.7	219	34.0	238	29.9
Poor	≤1.80	510	35.4	230	35.7	280	35.2
Mean ± SD		2.20 ± 0.65		2.17 ± 0.63		2.22 ± 0.67	
Aesthetics (1–4)^b^							
Good	2.80<	557	38.6	233	36.1	324	40.6
Fair	2.30<, ≤2.80	443	30.7	198	30.7	245	30.7
Poor	≤2.30	443	30.7	214	33.2	229	28.7
Mean ± SD		2.48 ± 0.67		2.42 ± 0.66		2.52 ± 0.66	
Traffic safety (1–4)^b^							
Good	3.00<	496	34.2	197	30.4	299	37.3
Fair	2.50<, ≤3.00	548	37.8	263	40.6	285	35.5
Poor	≤2.50	406	28.0	188	29.0	218	27.2
Mean ± SD		2.67 ± 0.54		2.63 ± 0.55		2.70 ± 0.54	
Crime safety (1–4)^b^							
Good	3.17<	585	40.3	267	41.2	318	39.6
Fair	2.83<, ≤3.17	445	30.7	211	32.6	234	29.1
Poor	≤2.83	421	29.0	170	26.2	251	31.3
Mean ± SD		2.97 ± 0.46		2.98 ± 0.45		2.96 ± 0.47	

^a^Classification of categories was by tertiles.

^b^Figures in parentheses indicate score ranges.

Table 3 shows the odds ratios for active walkers by environmental factor in the overall sample. Four environmental variables (high residential density, fair land use mix–diversity, good walking/cycling facilities, and good aesthetics) were significantly associated with neighborhood walking. Participants were more likely to walk when they perceived that there was high residential density (odds ratio, 1.47; 95% confidence interval, 1.11–1.96), fair land use mix–diversity (1.37, 1.04–1.81), good walking/cycling facilities (1.56, 1.19–2.04), and good aesthetics (1.49, 1.14–1.95). Regarding walking for particular purposes, there were specific associations between environment and walking. Active walking for daily errands was associated with 6 categories in 4 environmental variables: high residential density, good and fair land use mix–diversity, good and fair land use mix–access, and good street connectivity. In contrast, the environmental factors that were significantly associated with walking for leisure were different, and included good walking/cycling facilities, good and fair aesthetics, and good and fair traffic safety. The results regarding commuting on foot to work were similar to those for walking for daily errands: 3 environmental variables were significant—high residential density, good land use mix–diversity, and good land use mix–access.

**Table 3.** Odds ratios for active walkers by environmental factors (all respondents)

	Neighborhood walking			Walking for daily errands			Walking for leisure			Commuting on foot to work		
	*n* = 1443			*n* = 1461			*n* = 1461			*n* = 1083^e^		
				
	% of active walkers^c,d^	OR^a^ (95% CI)	*P* value	% of active walkers^c,d^	OR^a^ (95% CI)	*P* value	% of active walkers^c,d^	OR^a^ (95% CI)	*P* value	% of active walkers^c,d^	OR^b^ (95% CI)	*P* value
Residential density												
High	57.6 (246/427)	1.47 (1.11, 1.96)	0.008	54.4 (235/432)	2.09 (1.56, 2.81)	<0.001	33.8 (146/432)	0.94 (0.70, 1.26)	0.677	51.1 (162/317)	1.99 (1.41, 2.81)	<0.001
Medium	49.4 (252/510)	1.12 (0.85, 1.46)	0.424	41.8 (215/514)	1.30 (0.98, 1.72)	0.067	35.4 (182/514)	1.02 (0.78, 1.35)	0.868	38.8 (149/384)	1.26 (0.90, 1.76)	0.171
Low	43.6 (216/495)	1.00		33.9 (170/502)	1.00		35.3 (177/502)	1.00		27.3 (102/373)	1.00	
Land use mix–diversity												
Good	54.1 (251/464)	1.19 (0.89, 1.60)	0.238	48.4 (228/471)	1.69 (1.25, 2.30)	<0.001	34.8 (164/471)	0.93 (0.68, 1.27)	0.643	47.6 (162/340)	1.51 (1.06, 2.16)	0.023
Fair	55.0 (264/480)	1.37 (1.04, 1.81)	0.027	46.2 (223/483)	1.53 (1.14, 2.05)	0.004	37.9 (183/483)	1.17 (0.88, 1.57)	0.278	39.1 (140/358)	1.05 (0.74, 1.49)	0.769
Poor	41.2 (195/473)	1.00		34.1 (164/481)	1.00		32.6 (157/481)	1.00		29.6 (108/365)	1.00	
Land use mix–access												
Good	56.2 (266/473)	1.33 (1.00, 1.78)	0.053	52.2 (250/479)	2.11 (1.56, 2.84)	<0.001	37.0 (177/479)	1.01 (0.75, 1.36)	0.944	47.6 (157/330)	1.68 (1.18, 2.38)	0.004
Fair	51.1 (247/483)	1.17 (0.89, 1.55)	0.257	43.8 (212/484)	1.55 (1.16, 2.06)	0.003	35.1 (170/484)	1.00 (0.75, 1.34)	0.988	38.0 (139/366)	1.14 (0.81, 1.60)	0.441
Poor	42.9 (204/475)	1.00		33.0 (160/485)	1.00		33.0 (160/485)	1.00		30.9 (116/376)	1.00	
Street connectivity												
Good	50.6 (219/433)	1.01 (0.77, 1.34)	0.924	47.0 (205/436)	1.43 (1.07, 1.91)	0.015	36.5 (159/436)	1.05 (0.79, 1.40)	0.750	36.7 (115/313)	0.98 (0.70, 1.39)	0.929
Fair	52.1 (279/536)	1.11 (0.85, 1.45)	0.440	45.0 (243/540)	1.28 (0.97, 1.68)	0.080	34.3 (185/540)	1.03 (0.79, 1.36)	0.811	44.1 (179/406)	1.31 (0.95, 1.80)	0.097
Poor	47.6 (215/452)	1.00		37.0 (171/462)	1.00		34.6 (160/462)	1.00		33.8 (117/346)	1.00	
Walking/cycling facilities												
Good	55.8 (261/468)	1.56 (1.19, 2.04)	0.001	46.9 (222/473)	1.26 (0.96, 1.65)	0.100	39.1 (185/473)	1.47 (1.11, 1.93)	0.006	42.0 (144/343)	1.36 (0.99, 1.88)	0.059
Fair	50.9 (230/452)	1.22 (0.93, 1.60)	0.150	43.1 (197/457)	1.13 (0.86, 1.49)	0.381	35.0 (160/457)	1.21 (0.92, 1.61)	0.177	41.4 (139/336)	1.19 (0.86, 1.65)	0.298
Poor	44.3 (223/503)	1.00		39.2 (200/510)	1.00		31.0 (158/510)	1.00		33.2 (129/389)	1.00	
Aesthetics												
Good	57.8 (318/550)	1.49 (1.14, 1.95)	0.004	48.1 (268/557)	1.28 (0.97, 1.69)	0.079	43.4 (242/557)	2.22 (1.66, 2.97)	<0.001	40.8 (162/397)	1.03 (0.74, 1.42)	0.882
Fair	46.7 (204/437)	0.99 (0.75, 1.31)	0.942	41.5 (184/443)	1.04 (0.78, 1.39)	0.774	34.3 (152/443)	1.57 (1.16, 2.12)	0.004	38.0 (127/334)	0.90 (0.65, 1.27)	0.561
Poor	43.6 (191/438)	1.00		37.7 (167/443)	1.00		25.1 (111/443)	1.00		36.1 (122/338)	1.00	
Traffic safety												
Good	54.0 (263/487)	1.02 (0.77, 1.35)	0.895	43.3 (215/496)	0.87 (0.65, 1.17)	0.356	39.3 (195/496)	1.48 (1.10, 2.00)	0.009	41.8 (150/359)	1.08 (0.77, 1.51)	0.675
Fair	49.1 (265/540)	0.93 (0.71, 1.22)	0.591	43.4 (238/548)	0.99 (0.75, 1.31)	0.949	36.7 (201/548)	1.39 (1.04, 1.86)	0.025	36.9 (146/396)	0.92 (0.66, 1.28)	0.631
Poor	46.4 (188/405)	1.00		41.1 (167/406)	1.00		27.3 (111/406)	1.00		36.1 (116/321)	1.00	
Crime safety												
Good	50.4 (293/581)	1.03 (0.79, 1.36)	0.816	43.2 (253/585)	1.05 (0.8, 1.39)	0.721	36.6 (214/585)	1.07 (0.81, 1.42)	0.618	40.5 (169/417)	1.22 (0.87, 1.69)	0.245
Fair	51.6 (225/436)	1.14 (0.86, 1.52)	0.366	42.7 (190/445)	1.05 (0.79, 1.41)	0.721	35.5 (158/445)	1.14 (0.85, 1.53)	0.375	37.1 (125/337)	0.91 (0.65, 1.28)	0.590
Poor	47.8 (199/416)	1.00		42.5 (179/421)	1.00		32.3 (136/421)	1.00		36.6 (118/322)	1.00	

Abbreviations: OR, odds ratio; CI, confidence interval.

^a^Odds ratios were calculated after adjustment for age, sex, location of residence, employment status, education, BMI, and self-rated health.

^b^Odds ratios were calculated after adjustment for age, sex, location of residence, education, BMI, and self-rated health.

^c^For the 4 respective categories, an active walker was defined as a respondent who reported neighborhood walking >90 min/week, walking for daily errands, walking for leisure, or walking to work.

^d^Figures in parentheses indicate (number of active walkers/number of participants in category).

^e^Commuting on foot to work was examined only among the 1083 participants who were employed.

Analyses stratified by sex (men, Table 4; women, Table 5) revealed some differences between men and women. Walking for daily errands and commuting on foot to work were associated with a higher number of environmental variables in women than in men. In men, there was no significant association between environment and commuting on foot to work. In the analyses of walking for leisure, the associations between environment and walking also differed by sex. Among men, those who perceived good and fair walking/cycling facilities, good aesthetics, and good traffic safety tended to walk for leisure; among women, high residential density, good land use mix–diversity, and good and fair aesthetics were significantly associated with this type of walking. An interesting unexpected result was that women who reported high residential density and good land use mix–diversity walked less for leisure.

**Table 4.** Odds ratios for active walkers by environmental factors (men)

	Neighborhood walking			Walking for daily errands			Walking for leisure			Commuting on foot to work		
	*n* = 649			*n* = 654			*n* = 654			*n* = 559^e^		
			
	% of active walkers^c,d^	OR^a^ (95% CI)	*P* value	% of active walkers^c,d^	OR^a^ (95% CI)	*P* value	% of active walkers^c,d^	OR^a^ (95% CI)	*P* value	% of active walkers^c,d^	OR^b^ (95% CI)	*P* value
Residential density												
High	54.2 (96/177)	1.47 (0.95, 2.27)	0.083	36.5 (65/178)	1.74 (1.09, 2.76)	0.020	37.6 (67/178)	1.56 (0.99, 2.47)	0.056	48.4 (75/155)	1.33 (0.81, 2.18)	0.264
Medium	42.9 (100/233)	0.87 (0.58, 1.31)	0.503	29.5 (69/234)	1.20 (0.77, 1.88)	0.419	28.2 (66/234)	0.84 (0.54, 1.30)	0.439	43.7 (86/197)	1.18 (0.74, 1.88)	0.486
Low	40.8 (95/233)	1.00		22.0 (52/236)	1.00		33.5 (79/236)	1.00		31.2 (63/202)	1.00	
Land use mix–diversity												
Good	50.5 (107/212)	1.36 (0.87, 2.14)	0.180	29.0 (62/214)	1.21 (0.73, 1.99)	0.457	36.9 (79/214)	1.53 (0.95, 2.48)	0.081	48.3 (86/178)	1.34 (0.79, 2.27)	0.280
Fair	51.2 (108/211)	1.67 (1.09, 2.58)	0.019	35.5 (75/211)	1.70 (1.07, 2.71)	0.026	33.6 (71/211)	1.58 (1.00, 2.51)	0.052	44.0 (80/182)	1.20 (0.73, 1.97)	0.475
Poor	35.0 (75/214)	1.00		21.2 (46/217)	1.00		28.6 (62/217)	1.00		29.6 (56/189)	1.00	
Land use mix–access												
Good	51.5 (104/202)	1.37 (0.88, 2.13)	0.162	35.8 (73/204)	1.88 (1.17, 3.02)	0.009	35.8 (73/204)	1.41 (0.88, 2.26)	0.155	48.2 (81/168)	1.07 (0.64, 1.80)	0.784
Fair	46.9 (100/213)	1.11 (0.73, 1.67)	0.633	29.6 (63/213)	1.42 (0.90, 2.24)	0.135	34.3 (73/213)	1.23 (0.79, 1.91)	0.369	37.5 (69/184)	0.71 (0.44, 1.16)	0.175
Poor	39.4 (89/226)	1.00		21.8 (50/229)	1.00		29.3 (67/229)	1.00		36.8 (74/201)	1.00	
Street connectivity												
Good	43.8 (84/192)	0.83 (0.54, 1.26)	0.381	27.6 (53/192)	1.05 (0.66, 1.66)	0.831	33.3 (64/192)	1.01 (0.65, 1.58)	0.965	36.6 (59/161)	0.71 (0.43, 1.16)	0.173
Fair	48.7 (113/232)	1.08 (0.72, 1.62)	0.701	33.5 (78/233)	1.42 (0.92, 2.18)	0.111	32.2 (75/233)	1.20 (0.78, 1.84)	0.415	46.3 (94/203)	1.06 (0.67, 1.68)	0.803
Poor	44.7 (96/215)	1.00		25.1 (55/219)	1.00		33.3 (73/219)	1.00		38.0 (71/187)	1.00	
Walking/cycling facilities												
Good	50.5 (98/194)	1.72 (1.13, 2.61)	0.011	29.7 (58/195)	1.10 (0.71, 1.71)	0.677	38.5 (75/195)	1.90 (1.22, 2.95)	0.005	42.7 (70/164)	1.25 (0.78, 2.00)	0.363
Fair	48.6 (106/218)	1.46 (0.98, 2.19)	0.066	31.1 (68/219)	1.16 (0.76, 1.77)	0.499	33.8 (74/219)	1.56 (1.01, 2.40)	0.045	43.2 (80/185)	1.07 (0.67, 1.71)	0.762
Poor	38.8 (88/227)	1.00		26.1 (60/230)	1.00		27.0 (62/230)	1.00		36.6 (74/202)	1.00	
Aesthetics												
Good	53.7 (124/231)	1.41 (0.93, 2.12)	0.102	33.9 (79/233)	1.36 (0.88, 2.11)	0.163	39.1 (91/233)	1.76 (1.13, 2.74)	0.013	46.3 (93/201)	1.24 (0.77, 1.99)	0.370
Fair	41.3 (81/196)	0.94 (0.62, 1.44)	0.785	26.3 (52/198)	0.96 (0.61, 1.51)	0.853	32.8 (65/198)	1.42 (0.90, 2.25)	0.128	38.2 (65/170)	0.97 (0.60, 1.58)	0.910
Poor	40.8 (87/213)	1.00		25.7 (55/214)	1.00		26.6 (57/214)	1.00		35.4 (64/181)	1.00	
Traffic safety												
Good	50.0 (97/194)	1.26 (0.81, 1.95)	0.303	26.4 (52/197)	0.76 (0.47, 1.21)	0.245	38.6 (76/197)	1.65 (1.03, 2.64)	0.039	44.2 (72/163)	1.19 (0.72, 1.97)	0.487
Fair	47.5 (124/261)	1.18 (0.78, 1.78)	0.426	30.0 (79/263)	0.95 (0.62, 1.46)	0.817	35.4 (93/263)	1.48 (0.95, 2.32)	0.086	40.4 (90/223)	1.04 (0.65, 1.66)	0.877
Poor	38.3 (72/188)	1.00		28.7 (54/188)	1.00		23.9 (45/188)	1.00		35.7 (60/168)	1.00	
Crime safety												
Good	42.9 (114/266)	0.83 (0.55, 1.27)	0.400	25.8 (69/267)	0.67 (0.43, 1.05)	0.081	35.6 (95/267)	1.35 (0.85, 2.13)	0.201	40.5 (92/227)	1.00 (0.62, 1.62)	0.999
Fair	49.5 (103/208)	1.10 (0.71, 1.70)	0.682	28.9 (61/211)	0.77 (0.49, 1.21)	0.261	35.1 (74/211)	1.47 (0.92, 2.37)	0.108	38.0 (68/179)	0.71 (0.43, 1.18)	0.191
Poor	45.0 (76/169)	1.00		32.4 (55/170)	1.00		26.5 (45/170)	1.00		41.9 (62/148)	1.00	

Abbreviations: OR, odds ratio; CI, confidence interval.

^a^Odds ratios were calculated after adjustment for age, sex, location of residence, employment status, education, BMI, and self-rated health.

^b^Odds ratios were calculated after adjustment for age, sex, location of residence, education, BMI, and self-rated health.

^c^For the 4 respective categories, an active walker was defined as a respondent who reported neighborhood walking >90 min/week, walking for daily errands, walking for leisure, or walking to work.

^d^Figures in parentheses indicate (number of active walkers/number of participants in category).

^e^Commuting on foot to work was examined only among the 559 participants who were employed.

**Table 5.** Odds ratios for active walkers by environmental factors (women)

	Neighborhood walking			Walking for daily errands			Walking for leisure			Commuting on foot to work		
	*n* = 794			*n* = 807			*n* = 807			*n* = 524^e^		
				
	% of active walkers^c,d^	OR^a^ (95% CI)	*P* value	% of active walkers^c,d^	OR^a^ (95% CI)	*P* value	% of active walkers^c,d^	OR^a^ (95% CI)	*P* value	% of active walkers^c,d^	OR^b^ (95% CI)	*P* value
Residential density												
High	60.0 (150/250)	1.49 (1.02, 2.18)	0.038	66.9 (170/254)	2.35 (1.60, 3.43)	<0.001	31.1 (79/254)	0.64 (0.43, 0.96)	0.029	53.7 (87/162)	3.29 (1.97, 5.49)	<0.001
Medium	54.9 (152/277)	1.35 (0.93, 1.95)	0.111	52.1 (146/280)	1.32 (0.92, 1.90)	0.127	41.4 (116/280)	1.12 (0.77, 1.62)	0.566	33.7 (63/187)	1.45 (0.87, 2.40)	0.153
Low	46.2 (121/262)	1.00		44.4 (118/266)	1.00		36.8 (98/266)	1.00		22.8 (39/171)	1.00	
Land use mix–diversity												
Good	57.1 (144/252)	1.10 (0.74, 1.63)	0.643	64.6 (166/257)	2.14 (1.44, 3.17)	<0.001	33.1 (85/257)	0.63 (0.41, 0.95)	0.027	46.9 (76/162)	1.77 (1.07, 2.94)	0.026
Fair	58.0 (156/269)	1.21 (0.84, 1.76)	0.310	54.4 (148/272)	1.38 (0.95, 1.99)	0.092	41.2 (112/272)	0.96 (0.65, 1.40)	0.822	34.1 (60/176)	1.01 (0.61, 1.67)	0.960
Poor	46.3 (120/259)	1.00		44.7 (118/264)	1.00		36.0 (95/264)	1.00		29.5 (52/176)	1.00	
Land use mix–access												
Good	59.8 (162/271)	1.35 (0.91, 1.98)	0.131	64.4 (177/275)	2.28 (1.55, 3.35)	<0.001	37.8 (104/275)	0.78 (0.52, 1.16)	0.216	46.9 (76/162)	2.83 (1.67, 4.80)	<0.001
Fair	54.4 (147/270)	1.22 (0.84, 1.78)	0.298	55.0 (149/271)	1.63 (1.12, 2.36)	0.010	35.8 (97/271)	0.80 (0.54, 1.17)	0.249	38.5 (70/182)	1.98 (1.19, 3.29)	0.008
Poor	46.2 (115/249)	1.00		43.0 (110/256)	1.00		36.3 (93/256)	1.00		24.0 (42/175)	1.00	
Street connectivity												
Good	56.0 (135/241)	1.19 (0.81, 1.75)	0.364	62.3 (152/244)	1.78 (1.22, 2.60)	0.003	38.9 (95/244)	1.08 (0.73, 1.59)	0.704	36.8 (56/152)	1.28 (0.77, 2.13)	0.336
Fair	54.6 (166/304)	1.14 (0.80, 1.63)	0.478	53.7 (165/307)	1.20 (0.85, 1.71)	0.307	35.8 (110/307)	0.97 (0.67, 1.40)	0.857	41.9 (85/203)	1.61 (1.01, 2.57)	0.048
Poor	50.2 (119/237)	1.00		47.7 (116/243)	1.00		35.8 (87/243)	1.00		28.9 (46/159)	1.00	
Walking/cycling facilities												
Good	59.5 (163/274)	1.53 (1.07, 2.18)	0.020	59.0 (164/278)	1.35 (0.95, 1.91)	0.091	39.6 (110/278)	1.24 (0.87, 1.79)	0.239	41.3 (74/179)	1.54 (0.97, 2.43)	0.065
Fair	53.0 (124/234)	1.08 (0.75, 1.57)	0.669	54.2 (129/238)	1.09 (0.76, 1.57)	0.636	36.1 (86/238)	1.02 (0.70, 1.49)	0.928	39.1 (59/151)	1.40 (0.87, 2.26)	0.171
Poor	48.9 (135/276)	1.00		50.0 (140/280)	1.00		34.3 (96/280)	1.00		29.4 (55/187)	1.00	
Aesthetics												
Good	60.8 (194/319)	1.59 (1.10, 2.30)	0.013	58.3 (189/324)	1.24 (0.87, 1.77)	0.239	46.6 (151/324)	2.83 (1.90, 4.22)	<0.001	35.2 (69/196)	0.79 (0.49, 1.27)	0.335
Fair	51.0 (123/241)	1.02 (0.7, 1.5)	0.914	53.9 (132/245)	1.10 (0.76, 1.60)	0.613	35.5 (87/245)	1.69 (1.11, 2.57)	0.014	37.8 (62/164)	0.87 (0.54, 1.42)	0.578
Poor	46.2 (104/225)	1.00		48.9 (112/229)	1.00		23.6 (54/229)	1.00		36.9 (58/157)	1.00	
Traffic safety												
Good	56.7 (166/293)	0.82 (0.56, 1.20)	0.317	54.5 (163/299)	0.95 (0.65, 1.37)	0.768	39.8 (119/299)	1.26 (0.85, 1.87)	0.248	39.8 (78/196)	0.95 (0.59, 1.53)	0.835
Fair	50.5 (141/279)	0.72 (0.49, 1.04)	0.083	55.8 (159/285)	1.02 (0.70, 1.47)	0.928	37.9 (108/285)	1.23 (0.83, 1.82)	0.299	32.4 (56/173)	0.80 (0.49, 1.30)	0.372
Poor	53.5 (116/217)	1.00		51.8 (113/218)	1.00		30.3 (66/218)	1.00		36.6 (56/153)	1.00	
Crime safety												
Good	56.8 (179/315)	1.23 (0.85, 1.76)	0.272	57.9 (184/318)	1.41 (0.99, 2.01)	0.059	37.4 (119/318)	0.96 (0.67, 1.40)	0.844	40.5 (77/190)	1.35 (0.85, 2.16)	0.208
Fair	53.5 (122/228)	1.14 (0.78, 1.66)	0.504	55.1 (129/234)	1.28 (0.88, 1.86)	0.190	35.9 (84/234)	0.98 (0.66, 1.44)	0.909	36.1 (57/158)	1.02 (0.63, 1.66)	0.930
Poor	49.8 (123/247)	1.00		49.4 (124/251)	1.00		36.3 (91/251)	1.00		32.2 (56/174)	1.00	

Abbreviations: OR, odds ratio; CI, confidence interval.

^a^Odds ratios were calculated after adjustment for age, sex, location of residence, employment status, education, BMI, and self-rated health.

^b^Odds ratios were calculated after adjustment for age, sex, location of residence, education, BMI, and self-rated health.

^c^For the 4 respective categories, an active walker was defined as a respondent who reported neighborhood walking >90 min/week, walking for daily errands, walking for leisure, or walking to work.

^d^Figures in parentheses indicate (number of active walkers/number of participants in category).

^e^Commuting on foot to work was examined only among the 524 participants who were employed.

## DISCUSSION

In the present study, the perceived environmental features of a neighborhood were associated with walking in that neighborhood. In addition, the environmental variables associated with walking differed with regard to the purpose for walking, which was consistent with previous studies.^10^^,^^11^ Walking for transportation (ie, errands and commuting to work) was associated with neighborhood walkability, as defined by high residential density, mixed land use, and good street connectivity. Walking for leisure was associated with the quality of pedestrian facilities, neighborhood aesthetics, and traffic safety.

Because sex differences in the associations between environment and physical activity have not been widely studied, those observed in the present study are of particular interest. Sex-specific analyses revealed significant associations between environment and commuting on foot to work only in women. The reasons for this are unclear. One possible reason is that women are more likely to work within walking distance. The association between environment and walking for daily errands was also stronger and more consistent in women than in men, most likely because women play a greater role in managing households, and have more opportunities to walk for errands such as shopping, than do men. Because of this, neighborhood features may have been more important for this type of walking in women than in men.

There were some unexpected findings in women. High residential density and good land use mix–diversity were both associated with less leisure walking among women. These results have 2 implications. One possibility is that high residential density and good land use mix–diversity, which were consistently related to walking for transportation in previous studies,^11^ might create a less desirable environment for leisure walking. Leisure walking is generally faster and more continuous than transport walking. Very high residential density and a good land use mix could generate excess car and pedestrian traffic, thereby interfering with leisure walking. These results were not observed in studies conducted in the United States and Australia, probably because residential density is usually lower and land use mix is less diverse in these countries. We find it interesting that a particular environmental feature could promote 1 type of walking while inhibiting another. This finding also confirms the importance of examining purpose-specific walking in environmental studies. The second implication of the abovementioned findings is that styles of leisure walking might differ by sex. For example, women walking for leisure might seek out relaxing places and avoid high-density areas and mixed-use environments in order to escape people and distractions, while men may prefer more densely populated neighborhoods and convenient places for leisure walking, perhaps because they are not adversely affected by these environmental characteristics.

In a meta-analysis of 16 studies, Duncan reported that 4 environmental factors—physical activity facilities, sidewalks, shops and services (a variable similar to land use mix–diversity in the present study), and traffic safety—were associated with physical activity.^28^ Owen reviewed 18 studies that examined environmental correlates of walking and observed that aesthetic attributes, facilities for walking (sidewalks, trails), accessibility of destinations (similar to land use mix–diversity in this study), perception of traffic, and busy roads were associated with walking for particular purposes.^10^ This review also found that environmental factors associated with walking for exercise/leisure were different from those associated with walking for transport. Saelens and Handy showed that the findings from previous studies were confirmed in more recent investigations.^11^ Although the present study is the first to find that high residential density and mixed land use could interfere with leisure walking among women, our results were generally consistent with those of earlier studies. Thus, results regarding the environmental correlates of walking and the specific environmental associations with different purposes for walking are generalizable to the Japanese population. This is an important finding because the physical and cultural environments in Japan differ from those of the Western countries in which previous studies were conducted. Among Japanese adults, living in walkable communities, as defined by high residential density, good land use mix, and good street connectivity, is an important factor in walking for transport, while walking facilities (eg, sidewalks), aesthetics, and traffic safety are important factors in walking for leisure. These are robust findings across countries.

The results regarding crime safety have been inconsistent. In Duncan’s meta-analysis, no significant association was observed between crime safety and physical activity.^28^ However, some previous studies reported associations between crime safety and physical activity,^29^^,^^30^ and differences between sexes in these associations. Specifically, crime safety was associated with physical activity among women. We, too, examined sex-specific associations between perception of crime safety and walking; however, no significant association was identified for either sex. In Japan, variations in the perception of crime safety may be insufficient to demonstrate associations, as the country is generally perceived to be safe. Studies in a wider range of environments might more clearly illuminate the relationship between crime and physical activity.

There are several limitations in this study. First, the study was cross-sectional, so we are unable to address the direction of causality. Longitudinal or intervention studies are therefore needed in future research. Second, both environmental and walking measures were based on self-reports. We acknowledge the possibility of a discrepancy between perception and reality, even though the measures have been validated.^21^^–^^23^ Third, the response rate was somewhat low, which might have resulted in selection bias. If we assume that these participants tended to have healthier lifestyles and greater motivation and skills to overcome environmental barriers to walking, as compared with the general population, then they may walk regularly even in a poor environment. If so, this study would underestimate the association of environmental factors with walking behavior. Studies with a higher response rate and less selection bias will enhance rigor in this field of research. Fourth, participants lived in central and western Japan, not in the colder northern region of the country. Climate may be an independent determinant of walking or an effect modifier of the associations between environment and walking. To ascertain the generalizability of the findings, studies encompassing a wider range of environments are needed.

In spite of these limitations, the present study offers new evidence on physical activity and environment in Japan, and helps to fill a large gap in the data from non-Western countries. The results revealed specific environment—walking relationships and contributed to understanding the environmental correlates of our most common physical activity—walking.

### Conclusion

The association of neighborhood environment with walking differed by the purpose for walking. The results of the present study were generally consistent with those of studies conducted in Western countries. However, there were some differences, eg, high residential density and good land use mix were associated with less leisure walking among Japanese women. The findings suggest possible targets for interventions that aim to promote walking.

**Appendix.** Sample items on the Neighborhood Environment Walkability Scale–Abbreviated Japanese Version

Environmental factors	Number of items	Score range	Sample items	Choices
Residential density	5	5–805	How common are detached single-family residences in your immediate neighborhood?	1. None 2. A few 3. Some 4. Most 5. All
			How common are apartments or condos of 1–3 stories in your immediate neighborhood?	

Land use mix–diversity	23	1–5	About how long would it take to get from your home to the nearest businesses or facilities listed below if you walked to them? Please put only one check mark for each business or facility. ​ -convenience/small grocery store ​ -elementary school ​ -bank/credit union ​ -park	1. 1–5 min 2. 6–10 min 3. 11–20 min 4. 20–30 min 5. 30+ min 6. don’t know

Land use mix–access	6	1–4	Stores are within easy walking distance of my home.	1. strongly disagree 2. somewhat disagree 3. somewhat agree 4. strongly agree
			There are many places to go within easy walking distance of my home.	

Street connectivity	3	1–4	The distance between intersections in my neighborhood is usually short (100 yards or less; the length of a football field or less).	
			There are many alternative routes for getting from place to place in my neighborhood. (I don’t have to go the same way every time.)	

Walking/cycling facilities	4	1–4	There are sidewalks on most of the streets in my neighborhood.	
			There is a grass/dirt strip that separates the streets from the sidewalks in my neighborhood.	

Aesthetics	4	1–4	There are many attractive natural sights in my neighborhood (such as landscaping, views).	
			There are attractive buildings/homes in my neighborhood.	

Traffic safety	4	1–4	There is so much traffic along nearby streets that it makes it difficult or unpleasant to walk in my neighborhood.
			The speed of traffic on most nearby streets is usually slow (30 mph or less).

Crime safety	5	1–4	My neighborhood streets are well lit at night.
			Walkers and bikers on the streets in my neighborhood can be easily seen by people in their homes.
